# Development of the Italian clinical practice guideline on diagnosing and treating obesity in adults: scope and methodological aspects

**DOI:** 10.1007/s40519-025-01747-y

**Published:** 2025-06-04

**Authors:** Rocco Barazzoni, Silvio Buscemi, Luca Busetto, Paolo Sbraccia, Simona Bo, Emanuele Cereda, Marco Chianelli, Sonja Chiappetta, Riccardo Dalle Grave, Walter de Caro, Giovanni Docimo, Giuseppe Galloro, Primiano Iannone, Frida Leonetti, Fabrizia Lisso, Maria Caterina Manca, Gerardo Medea, Manuela Merli, Anna Maria Moretti, Giuseppe Navarra, Uberto Pagotto, Barbara Paolini, Giovanni Papa, Nicola Perrotta, Andrea Pession, Vincenzo Pilone, Vincenzo Provenzano, Cecilia Ricciardi Rizzo, Maurizio Santomauro, Cristina Segura Garcia, Federico Spandonaro, Samir Sukkar, Patrizia Todisco, Dario Tuccinardi, Andrea Vania, Valentina Vanzi, Riccardo Williams, Iris Zani, Benedetta Ragghianti, Giovanni Antonio Silverii, Matteo Monami, the panel and Evidence Review Team for the Italian Guidelines on Surgical Treatment of Obesity

**Affiliations:** 1 Department of Medical, Surgical and Health Sciences, University of Trieste, Trieste, Italy; 2 Department of Promozione della Salute, Materno-Infantile, Medicina Interna e Specialistica di Eccellenza (PROMISE), University of Palermo, Palermo, Italy; 3 Department of Medicine, University of Padova, Padua, Italy; 4 Department of Systems Medicine, University of Rome Tor Vergata, Rome, Italy; 5 Department of Medical Sciences, ASO Città della Salute e della Scienza Hospital, University of Torino, Torino, Italy; 6 SC Dietetica e Nutrizione Clinica Fondazione, IRCCS Policlinico San Matteo, Pavia, Italy; 7 Regina Apostolorum Hospital Albano, Rome, Italy; 8 UOC Chirurgia Generale e Laparoscopica Ospedale Evangelico Betania, Naples, Italy; 9 Department of Eating and Weight Disorders, Villa Garda Hospital, Garda, Italy; 10 Sapienza Università di Roma, Rome, Italy; 11 University of Campania “Luigi Vanvitelli”, Caserta, Italy; 12 Department of Clinical Medicine and Surgery Unit of Surgical, Endoscopy University of Naples Federico II - School of Medicine, Naples, Italy; 13 UOC Medicina Interna C, Ospedale Maggiore, Bologna, Italy; 14 Sapienza University of Rome, SM Goretti Hospital, Latin, Italy; 15 Sant’Anna Hospital, Como, Italy; 16 UOS Medicina Legale Centro, Azienda USL di Bologna, Bologna, Italy; 17 Medico di Medina Generale ASST Garda, Brescia, Italy; 18 Department of Translation and Precision Medicine, Policlinico Universitario Umberto 1, Rome, Italy; 19 UOC Pneumologia, Università di Bari, Bari, Italy; 20 DU di Patologia Umana DETEV, Università di Messina, Messina, Italy; 21 IRCCS Azienda Ospedaliero-Universitaria di Bologna, Bologna, Italy; 22 Dietetica e Nutrizione Clinica, Azienda Ospedaliera Universitaria Senese, Siena, Italy; 23 Head of Plastic Surgery Unit and Plastic Reconstructive and Aesthetic Surgery, University of Trieste, Trieste, Italy; 24 Azienda Ospedaliera Regionale “San Carlo”, Ospedale di Villa d’Agri, Potenza, Italy; 25 Dipartimento di Scienze Mediche e Chirurgiche, Università di Bologna, Bologna, Italy; 26 Dipartimento di Sanità Pubblica, Scuola Medicina e Chirurgia, Università Degli Studi Di Napoli “Federico II”, Naples, Italy; 27 Istituto e Clinica s Chiara, Partinico, PA Italy; 28 Department of Public Health, Experimental and Forensic Medicine, University of Pavia, Pavia, Italy; 29 Department of Advanced Biomedical Sciences, University of Naples Federico II, Naples, Italy; 30 Magna Græcia University of Catanzaro, Catanzaro, Italy; 31 Dipartimento di Economia e Istituzioni, University of Rome Tor Vergata, Rome, Italy; 32 U.O. Dietetica e Nutrizione Clinica, IRCCS Ospedale Policlinico San Martino di Genova, Genova, Italy; 33 Psychonutritional Center, Verona, Italy; 34 Fondazione Policlinico Universitario Campus Bio-Medico, Rome, Italy; 35 Independent Researcher, Rome, Italy; 36 Centro Interdipartimentale per la Ricerca e la Formazione, Università degli Studi di Roma Tor Vergata, Rome, Italy; 37 Dipartimento di Psicologia Dinamica e Clinica, Università degli Studi di Roma “Sapienza”, Rome, Italy; 38 Amici Obesi Onlus, Milan, Italy; 39 Diabetology and Metabolic Disease Unit, Careggi Teaching Hospital and University of Florence, Largo Brambilla 3, 50141 Florence, Italy

**Keywords:** Obesity, Guidelines, GRADE, Methods

## Abstract

Overweight and obesity are substantial, growing public health concerns due to their huge direct and indirect negative impact on health. Obesity-associated complications and comorbid conditions include metabolic, cardiovascular, renal, liver and respiratory diseases, cancers, and functional limitations, leading to higher all-cause and cardiovascular mortality, and incident disability. The development of rigorous guidelines considering and comparing all possible therapeutic strategies is of critical importance, and a relevant tool for improving the quality of care and increasing the appropriateness of therapeutic choices. The Italian National Institute of Health (ISS—Istituto Superiore di Sanità) appointed the Italian Obesity Society (SIO—Società Italiana dell’Obesità) and other key scientific societies with a relevant stakeholder role on the theme issue to design and develop a new Italian guideline for the management of obesity in adult subjects, aimed at assisting healthcare professionals in the consideration of lifestyle, pharmacological, endoscopic, and surgical options for the treatment of overweight and obesity, as well as related conditions. We adopted Grading of Recommendations, Assessment, Development and Evaluations (GRADE) methodology, strongly endorsed by Istituto Superiore di Sanità to develop trustworthy guidelines to be accepted onto Sistema Nazionale Linee Guida, the reference repository of national clinical practice guidelines for the Servizio Sanitario Nazionale.

**Supplementary Information:**

The online version contains supplementary material available at 10.1007/s40519-025-01747-y.

## Introduction

Overweight and obesity are growing public health concerns due to their huge direct and indirect negative impact on health. Obesity often begins in childhood or adolescence, although it can also manifest in adulthood. This chronic condition results from an intricate interplay of genetic, environmental, and behavioral factors. Genetic predisposition can influence one's susceptibility to weight gain, while environmental factors such as sedentary lifestyles and high-calorie diets further exacerbate the risk. Excess in adipose tissues can subsequently contribute to metabolic diseases and several obesity-associated medical conditions which can negatively affect the prognosis of subjects affected by “preclinical” obesity [1]. Systemic obesity-associated medical conditions affect all organs and include metabolic, cardiovascular, renal, liver, and respiratory diseases, cancer, and functional limitations, leading to higher all-cause and cardiovascular mortality, and incident disability. It has been estimated that half of the excess risk for coronary heart disease and about three-quarters of the excess risk for stroke was mediated through obesity-associated high blood pressure, cholesterol, and glucose concentrations [2]. Aside from the health impact of excess body weight and fat, the related economic burden represents a major and growing issue for many countries [3].

The treatment of overweight and obesity includes lifestyle interventions (LSI), medications, and surgical options; all of them are commonly characterized by limited long-term efficacy and/or few available data on their effectiveness and safety [4].

Metabolic bariatric surgery (MBS), which has been developed for achieving a relevant weight loss above 20–25% of initial body weight [5], has also been shown to have a therapeutic potential for reducing obesity-related complications, such as hypertension [6], type 2 diabetes [6, 7] and obstructive sleep apnea [8]. However, the use of surgical approaches has been limited by organizational and economic issues.

Several national and international guidelines promoted by scientific societies, such as the European Association for the Study of Obesity (EASO) [9], the European Society for Clinical Nutrition and Metabolism (ESPEN) [10], the American Gastroenterological Association (AGA) [11], the American Society for Metabolic and Bariatric Surgery (ASMBS) [12], the International Federation for the Surgery of Obesity and Metabolic Disorders (IFSO) [13], the Associazione Medici Endocrinologi (AME) [14] and the Italian Society of Bariatric and Metabolic Surgery for Obesity (SICOB) [5], have proposed several therapeutic algorithms reflecting their main expertise (i.e., lifestyle [10], pharmacological [9, 11, 13], or surgical [5, 12] approaches, respectively). However, the treatment of obesity often requires a multi-professional and multimodal approach [14], not fully adopted by the current guidelines; the development of a GRADE-based guideline considering and comparing all of the possible therapeutic strategies might improve the quality and the appropriateness of care.

For all the above-mentioned reasons, the Italian National Institute of Health (ISS—Istituto Superiore di Sanità), entitled by Italian Law and the Ministry of Health to assess and publish trustworthy guidelines, entrusted the Società Italiana dell’Obesità (SIO) and other key scientific societies to develop a new Italian guideline for the management of obesity in adults. This guideline is aimed at assisting healthcare professionals involved in the management of patients living with overweight/obesity. In the Italian national legal environment [15], the inclusion of guidelines in the National Guideline System is possible only after a careful methodological and formal revision by the National Center for Clinical Excellence of the Ministry of Health. In the development of national guidelines, the Center for Clinical Excellence recommends the use of Grading of Recommendations, Assessment, Development, and Evaluations (GRADE) methodology [16], which requires the explicit, preliminary identification of clear clinical questions as well as the definition of relevant outcomes for each question. The present paper reports on the steps followed for developing questions and the definition of outcomes for the new Italian guideline for the management of obesity.

## Methods

### Characteristics of the panel and evidence review team

Panel members, designed by SIO in collaboration with 35 Italian scientific societies indicated by the ISS (Table S1), elected a coordinator (RB) and nominated the members of the evidence review team (ERT), aimed at collecting and analyzing evidence, without participating in the definition of clinical questions, outcomes, and recommendations.

A detailed list of the 40 members of the panel, with their roles and affiliations, and of the 2 members of the ERT, is reported in Table S1. All members of the panel and the ERT compiled a declaration of conflicts of interest, collectively discussed to determine their relevance. In all cases, the reported conflicts were considered trivial and all components of the panel and the ERT were entitled to participate in the development of all recommendations.

### GRADE methodology for the development of guidelines

The GRADE method [16] was developed to limit the impact of panelists’ opinions and prejudices in formulating recommendations when developing a clinical guideline. The adherence to this stringent methodology should theoretically be of help in building recommendations based on the available evidence deriving from adequately designed peer-reviewed studies. The definition of a scoping document, illustrating aims, target population, and health professionals is the first task for the development of guidelines. The subsequent step consists of defining several clinical questions named PICO (Patient, Intervention, Comparison, Outcome) [16]; each recommendation is developed to give an appropriate answer to any question formulated by the panelists and approved by the panel. In this regard, the panel of experts has the task of defining for each PICO several potentially relevant clinical outcomes. Each outcome is then rated for importance and relevance by the panel (from 1 to 9). Outcomes receiving a rating of at least 7.0 are classified as “critical” and represent the basis for the development of the recommendation.

The task of the ERT is that of performing a systematic review and meta-analysis of any available relevant studies using predefined search strategies, inclusion criteria, and statistical analyses. Studies and related meta-analyses are assessed for methodological quality to verify the actual strength of available evidence. Economic evaluations (usually based on cost–utility ratio), organizational impact, equity, acceptability, and feasibility are other important components of GRADE methodology with a relevant impact on the strength of each recommendation, which should include all those elements.

The panelists decided to consider randomized controlled trials as the reference study design for all PICO, allowing the inclusion of nonrandomized studies only for clinical questions related to nonpharmacological treatments (i.e., education, diagnostic tools, etc.).

### Delphi process

A web-based Delphi method was used to define relevant clinical questions. Delphi methodology consists of a structured technique aimed at obtaining a consensus opinion from a panel of experts in areas, wherein evidence is scarce or conflicting [17].

Between September and December 2024, panelists were invited to propose PICO and to vote by expressing their level of agreement or disagreement on each proposed question. The vote was performed using a 5-point Likert scale, scored from 1 to 5 (1, strongly disagree; 2, disagree; 3, agree; 4, mostly agree; and 5, strongly agree) and a positive consensus was achieved only when more than 66% of panelists agree (from 3 to 5 points) about the relevance of the PICO. In case of more than 66% disagreement (from 1 to 2 points), the PICO was not considered relevant and, therefore, dismissed. When consensus was not reached (i.e., the sum for disagreement or agreement was below 66%) [18], panelists were asked to re-rate in a second round their agreement/disagreement, after internal discussion and potential modifications with all panelists.

## Results

The panel of experts was composed of 40 members (14 women, 35%) with a mean age of 57.0 ± 7.9 years. A detailed list of members along with their affiliation, tasks, and roles is reported in Table S1. One of the nominated members (AGIPPSA—Associazione Gruppi Italiani Psicoterapia Psicoanalitica dell’Adolescenza) formally declined to collaborate on the project and to vote on the proposed PICO and outcomes.

The guidelines will apply to adult (age > 18 years) patients affected by overweight or obesity (BMI ≥ 27 kg/m^2^). The setting of healthcare systems and human and financial resources across Italian regions will be considered for the development of the present guideline. Therefore, their applicability is primarily intended for the Italian National Health Care system and healthcare professionals (i.e., obesity experts, bariatric surgeons, general practitioners, nutrition experts, psychologists, internists, and endocrinologists/diabetologists).

The panel identified 14 clinical questions (PICO) and achieved an immediate consensus for all of them, with 13 approved and 1 rejected. The approved questions and their related critical (mean values ≥ 7.0) and non-critical (mean values < 7.0) outcomes are reported in Table 1. Only one PICO reported no critical outcomes and, therefore, excluded from the upcoming guidelines.

**Table 1:**
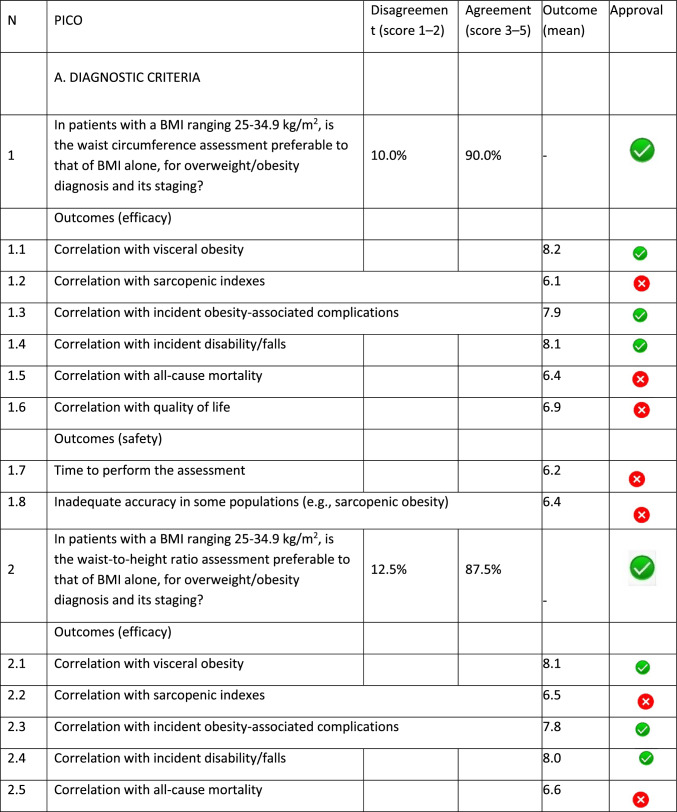

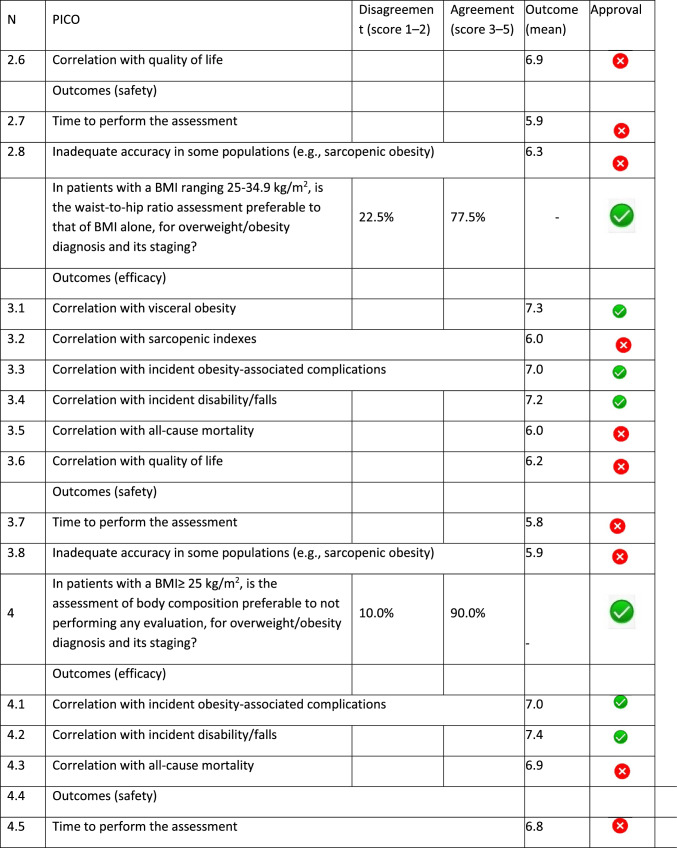

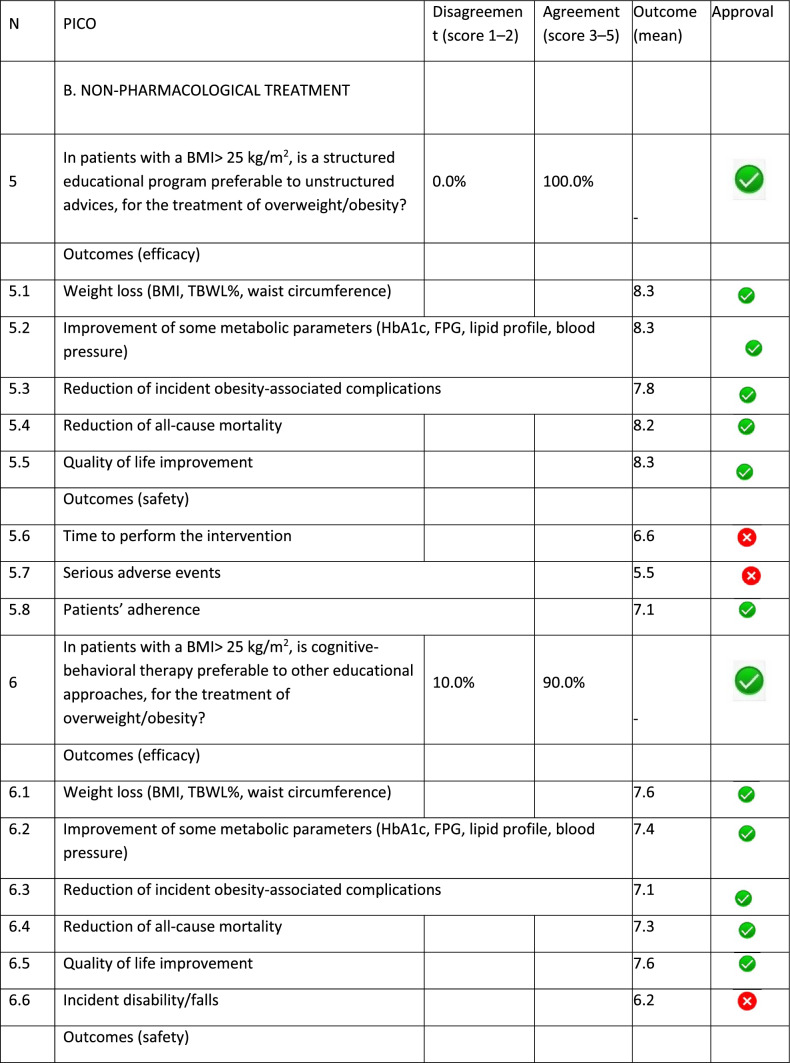

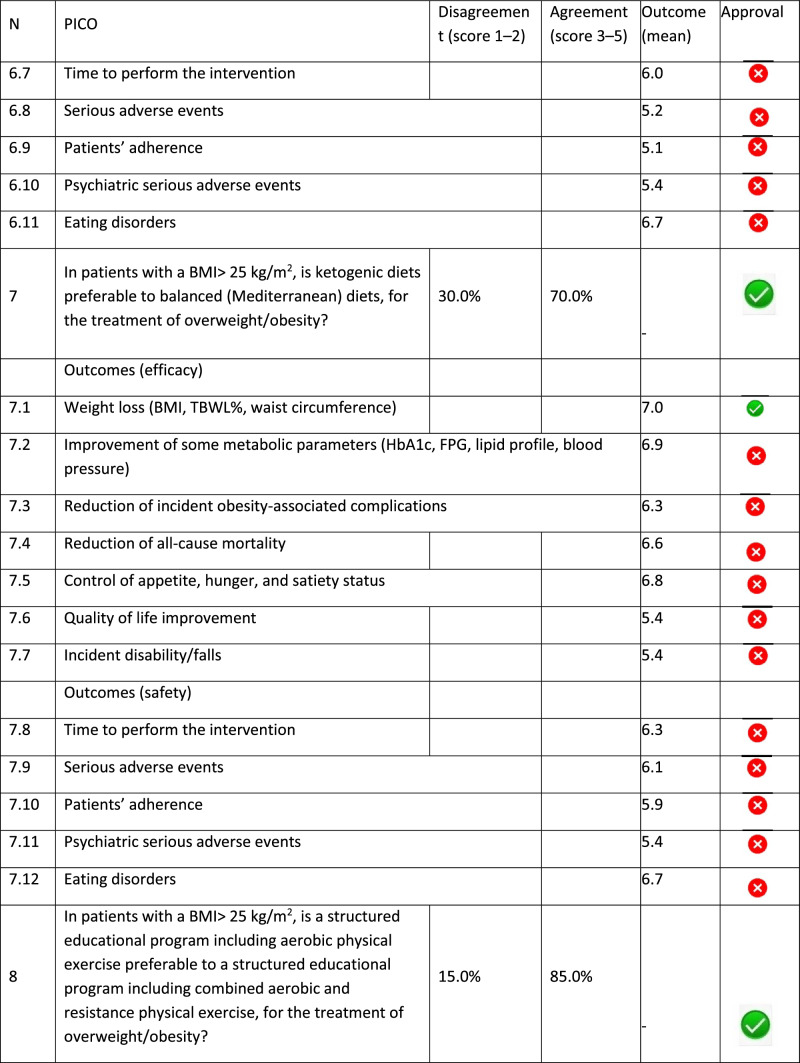

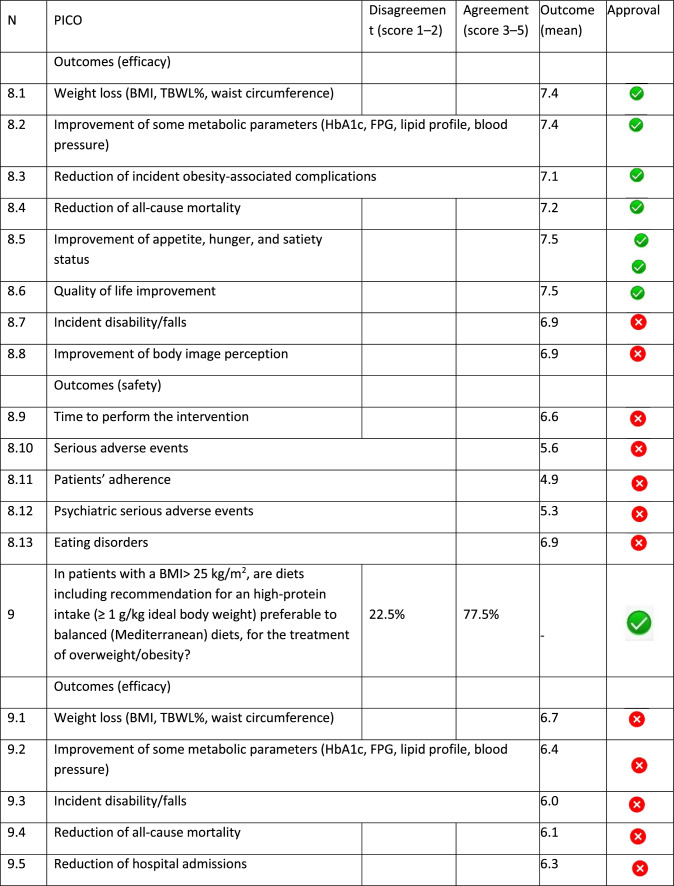

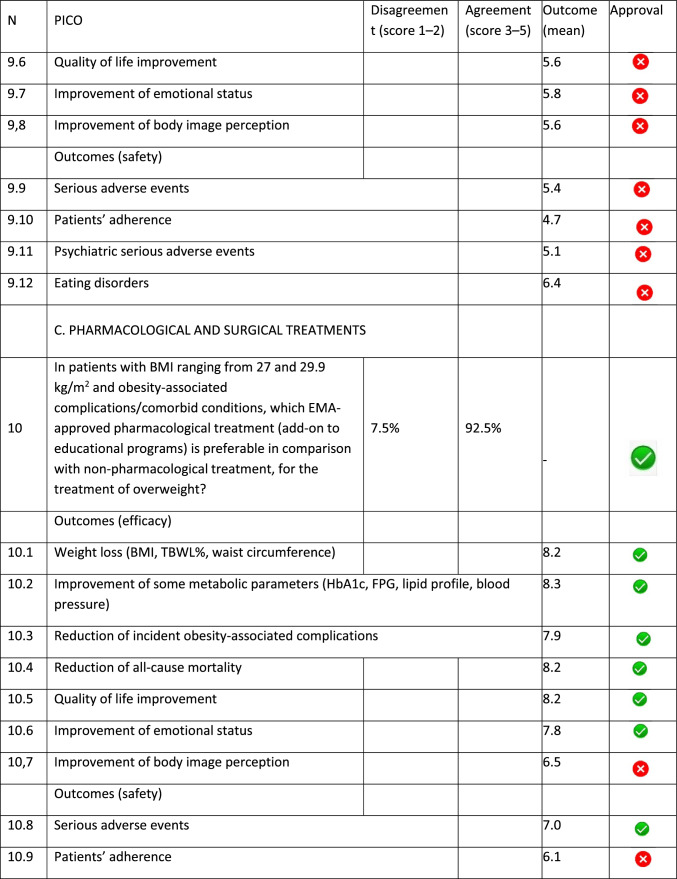

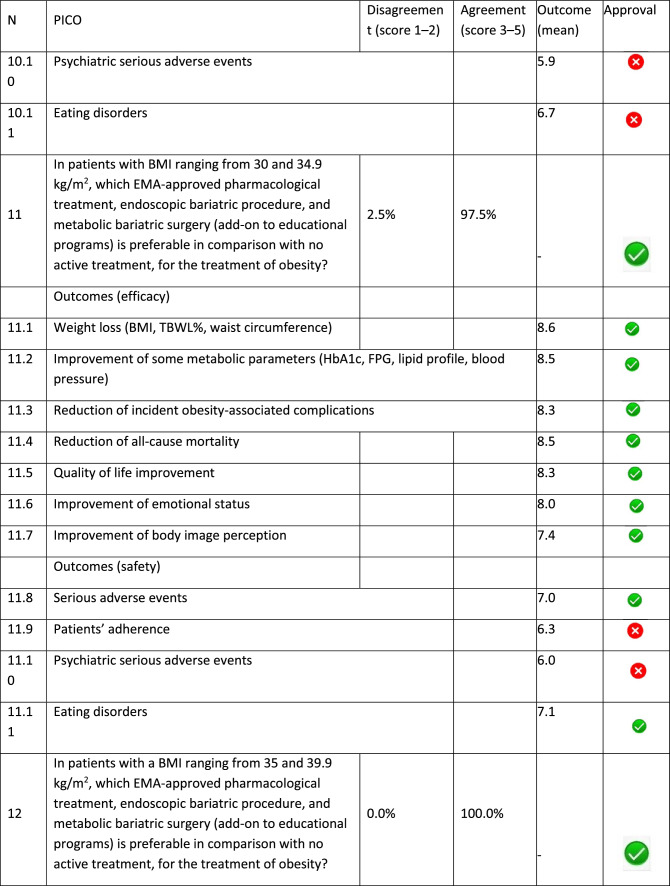

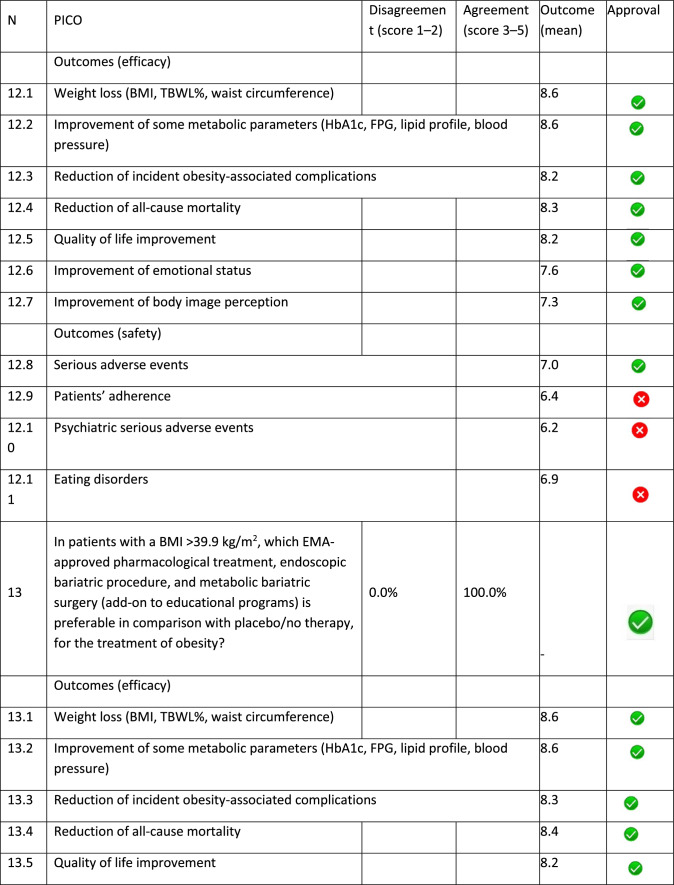

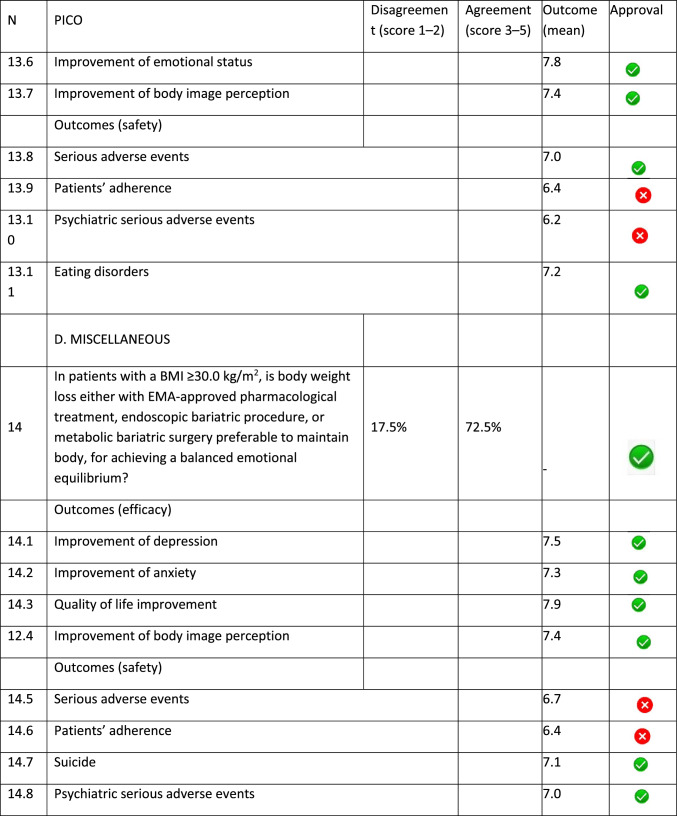
Delphi survey results and outcomes approval process. Green circle: approved; red circle: not approved

The 13 clinical questions approved were organized into four domains:

A. Diagnostic criteria (4 questions);
B. Nonpharmacological treatments (4 questions);
C. Pharmacological, endoscopic, and surgical treatments (4 questions);
D. Miscellaneous (1 question).

The evidence review team identified the characteristics of relevant studies for each PICO and critical outcomes, defining the search strategy and study inclusion and exclusion criteria. The search strategy used for all diagnostic PICO will be: “obesity AND (waist or waist-to-hip or waist-to-height or body composition or fat mass or fat-free mass)” restricting the search to “clinical studies”. The search strategy used for all therapeutic PICO will be: “obesity AND (orlistat or phentermine or ephedrine plus caffeine or phentermine plus topiramate or naltrexone plus bupropion or liraglutide or semaglutide or tirzepatide or Sleeve Gastrectomy or Roux en Y Gastric Bypass or One Anastomosis Gastric Bypass or Laparoscopic Adjustable Gastric Banding or Bilio-Pancreatic Diversion or Single Anastomosis Duodenal–Ileal bypass or Intragastric Balloons or Primary Obesity Surgery Endoluminal or Endoscopic Sleeve Gastroplasty or aspiration therapy or Duodenal–Jejunal Bypass Liner (DJBL) or lifestyle interventions)”, restricting the search to “randomized clinical trials”.

The expected start date for evidence research for all the included PICO is 15th February, 2025.

## Discussion

The areas covered by the clinical questions identified by panelists include indications for the appropriate use of diagnostic tools, such as waist circumference assessment, waist-to-hip and waist-to-height ratio calculation, medical nutritional therapy, and types of pharmacological and surgical treatments. The focus on diagnostic criteria and tools should not be surprising, and it is being recently widely debated: obesity reflects excessive fat deposits and some commonly used tools such as BMI (weight/height^2^) cannot provide reliable information for all subjects (i.e., underestimation of body fat excess in sarcopenic individuals or overestimation in fit subjects with high lean-muscle mass). Recent proposals formally advocate that excess adiposity should be further defined and confirmed by either direct measurement of body fat, or with anthropometric measurements (e.g., waist circumference, waist-to-hip ratio, or waist-to-height ratio) in addition to BMI [19].

Regarding medical nutritional therapy (MNT), a cornerstone of obesity management and treatment [20], the present guidelines will explore in adults living with obesity which NT approach is preferable (i.e., restrictive, ketogenic, and Mediterranean approach) and whether a combined aerobic and resistance physical exercise program can provide better results in terms of body weight reduction and metabolic control.

The choice of a surgical or a non-surgical approach for the treatment of obesity and related metabolic conditions is a complex issue, posing challenging concerns about the appropriateness of the therapeutic strategy for different patient groups. In addition, considering the current legislation on professional liability [15], correct identification of proper indications can support clinicians in an environment characterized by increasing frequency of legal claims and controversies. In patients referred to surgical treatment, the choice of the most appropriate intervention is also a major concern for surgeons; the collection and critical evaluation of available evidence from methodologically valid studies should represent a more appropriate support for this decision. Similar concerns are raised for the choice of one anti-obesity drug over the others. The decision should be only partially led by intrinsic efficacy in reducing body weight, also including patient phenotype (e.g., gender, social aspects, BMI target, presence of comorbid condition, such as OSAS, previous cardiovascular disease, etc.).

The panelists planning the development of these guidelines recognized the central role of longer-term hard outcomes, such as mortality, cardiovascular disease, malignancies, and control of pre-existing obesity-associate comorbid conditions. The availability of sufficient evidence for a reliable assessment of the effects of any anti-obesity strategy on those outcomes will be verified in the process of developing the present guidelines. Moreover, the choice of a specific therapeutic option should be based on an accurate assessment of the risk–benefit ratio, together with economic evaluations. This means that serious adverse events will be systematically and carefully collected and analyzed to rank all available treatments. Safety outcomes have been included for most clinical questions, concurring with the development of recommendations.

Transparency in developing a GRADE-based guideline is one of the major determinants of its quality [21]. The GRADE manual recommends the publication of clinical questions, relevant outcomes, and summaries of evidence for each outcome [22]. The panel of experts involved in the present project decided to go beyond these requirements, by preemptively reporting here *in extenso* the entire process leading to clinical questions and definition of critical outcomes. In addition, the search strategy and inclusion criteria for the systematic review and meta-analysis for each outcome have been described in the present study, thereby allowing for transparent reproducibility of the whole process. Notably, the panel also decided to extensively publish in peer-reviewed journals relevant systematic reviews and meta-analyses needed and generated for the formulation of the guideline.

## Supplementary Information

Below is the link to the electronic supplementary material.

Supplementary material 1.

## Data Availability

No datasets were generated or analysed during the current study.
