# Correction: Development of the Italian clinical practice guideline on diagnosing and treating obesity in adults: scope and methodological aspects

**DOI:** 10.1007/s40519-025-01776-7

**Published:** 2025-08-21

**Authors:** Rocco Barazzoni, Silvio Buscemi, Luca Busetto, Paolo Sbraccia, Simona Bo, Emanuele Cereda, Marco Chianelli, Sonja Chiappetta, Riccardo Dalle Grave, Walter de Caro, Giovanni Docimo, Giuseppe Galloro, Primiano Iannone, Frida Leonetti, Fabrizia Lisso, Maria Caterina Manca, Gerardo Medea, Manuela Merli, Anna Maria Moretti, Giuseppe Navarra, Uberto Pagotto, Barbara Paolini, Giovanni Papa, Nicola Perrotta, Andrea Pession, Vincenzo Pilone, Vincenzo Provenzano, Cecilia Ricciardi Rizzo, Maurizio Santomauro, Cristina Segura Garcia, Federico Spandonaro, Samir Sukkar, Patrizia Todisco, Dario Tuccinardi, Andrea Vania, Valentina Vanzi, Riccardo Williams, Iris Zani, Benedetta Ragghianti, Giovanni Antonio Silverii, Matteo Monami, the panel and Evidence Review Team for the Italian Guidelines on Surgical Treatment of Obesity

**Affiliations:** 1 Department of Medical, Surgical and Health Sciences, University of Trieste, Trieste, Italy; 2 Department of Promozione della Salute, Materno-Infantile, Medicina Interna e Specialistica di Eccellenza (PROMISE), University of Palermo, Palermo, Italy; 3 Department of Medicine, University of Padova, Padua, Italy; 4 Department of Systems Medicine, University of Rome Tor Vergata, Rome, Italy; 5 Department of Medical Sciences, ASO Città della Salute e della Scienza Hospital, University of Torino, Torino, Italy; 6 SC Dietetica e Nutrizione Clinica Fondazione, IRCCS Policlinico San Matteo, Pavia, Italy; 7 Regina Apostolorum Hospital Albano, Rome, Italy; 8 UOC Chirurgia Generale e Laparoscopica Ospedale Evangelico Betania, Naples, Italy; 9 Department of Eating and Weight Disorders, Villa Garda Hospital, Garda, Italy; 10 Sapienza Università di Roma, Rome, Italy; 11 University of Campania “Luigi Vanvitelli”, Caserta, Italy; 12 Department of Clinical Medicine and Surgery Unit of Surgical, Endoscopy University of Naples Federico II - School of Medicine, Naples, Italy; 13 UOC Medicina Interna C, Ospedale Maggiore, Bologna, Italy; 14 Sapienza University of Rome, SM Goretti Hospital, Latin, Italy; 15 Sant’Anna Hospital, Como, Italy; 16 UOS Medicina Legale Centro, Azienda USL di Bologna, Bologna, Italy; 17 Medico di Medina Generale ASST Garda, Brescia, Italy; 18 Department of Translation and Precision Medicine, Policlinico Universitario Umberto 1, Rome, Italy; 19 UOC Pneumologia, Università di Bari, Bari, Italy; 20 DU di Patologia Umana DETEV, Università di Messina, Messina, Italy; 21 IRCCS Azienda Ospedaliero-Universitaria di Bologna, Bologna, Italy; 22 Dietetica e Nutrizione Clinica, Azienda Ospedaliera Universitaria Senese, Siena, Italy; 23 Head of Plastic Surgery Unit and Plastic Reconstructive and Aesthetic Surgery, University of Trieste, Trieste, Italy; 24 Azienda Ospedaliera Regionale “San Carlo”, Ospedale di Villa d’Agri, Potenza, Italy; 25 Dipartimento di Scienze Mediche e Chirurgiche, Università di Bologna, Bologna, Italy; 26 Dipartimento di Sanità Pubblica, Scuola Medicina e Chirurgia, Università Degli Studi Di Napoli “Federico II”, Naples, Italy; 27 Istituto e Clinica s Chiara, Partinico, PA Italy; 28 Department of Public Health, Experimental and Forensic Medicine, University of Pavia, Pavia, Italy; 29 Department of Advanced Biomedical Sciences, University of Naples Federico II, Naples, Italy; 30 Magna Græcia University of Catanzaro, Catanzaro, Italy; 31 Dipartimento di Economia e Istituzioni, University of Rome Tor Vergata, Rome, Italy; 32 U.O. Dietetica e Nutrizione Clinica, IRCCS Ospedale Policlinico San Martino di Genova, Genova, Italy; 33 Psychonutritional Center, Verona, Italy; 34 Fondazione Policlinico Universitario Campus Bio-Medico, Rome, Italy; 35 Independent Researcher, Rome, Italy; 36 Centro Interdipartimentale per la Ricerca e la Formazione, Università degli Studi di Roma Tor Vergata, Rome, Italy; 37 Dipartimento di Psicologia Dinamica e Clinica, Università degli Studi di Roma “Sapienza”, Rome, Italy; 38 Amici Obesi Onlus, Milan, Italy; 39 Diabetology and Metabolic Disease Unit, Careggi Teaching Hospital and University of Florence, Largo Brambilla 3, 50141 Florence, Italy

**Correction to: Eating and Weight Disorders - Studies on Anorexia, Bulimia and Obesity (2025) 30:47** 10.1007/s40519-025-01747-y

In this article [1], the given and family name of the author Riccardo Dalle Grave was incorrectly structured. The name was displayed correctly in all versions at the time of publication.

The original article has been corrected.
