# Supplementary material for: Development of the Italian clinical practice guideline on diagnosing and treating obesity in adults: scope and methodological aspects
Source: Eat Weight Disord. 2025 Jun 4;30(1):47. doi: 10.1007/s40519-025-01747-y (PMC12133994; doi:10.1007/s40519-025-01747-y)
Supplement: Supplementary file 1 — Supplementary material 1. [file 40519_2025_1747_MOESM1_ESM.docx]

**Table S1 –** Characteristics and tasks of all panelists**.**

| N | Name | Role | Scientific society |
| --- | --- | --- | --- |
| 1 | Rocco Barazzoni | *Member* | SIO |
| 2 | Sonja Chiappetta | *Member* | ACOI |
| 3 | Barbara Paolini | *Member* | ADI |
| 4 | Francesca Tonucci | *Member* | AGIPPSA |
| 5 | Riccardo Dalle Grave | *Member* | AIDAP |
| 6 | Riccardo Williams | *Member* | AIP |
| 7 | Dario Tuccinardi | *Member* | AMD |
| 8 | Marco Chianelli | *Member* | AME |
| 9 | Iris Zani | *Member* | Amici Obesi |
| 10 | Valentina Vanzi | *Member* | ANIED |
| 11 | Cecilia Ricciardi Rizzo | *Member* | ANSISA |
| 12 | Fabrizia Lisso | *Member* | ASAND |
| 13 | Walter de Caro | *Member* | CNAI |
| 14 | Anna Maria Moretti | *Member* | GISEG |
| 15 | Nicola Perrotta | *Member* | SICE |
| 16 | Giuseppe Navarra | *Member* | SICOB |
| 17 | Giovanni Papa | Member | SICPRE |
| 18 | Frida Leonetti | *Member* | SID |
| 19 | Uberto Pagotto | *Member* | SIE |
| 20 | Giuseppe Galloro | *Member* | SIED |
| 21 | Manuela Merli | *Member* | SIGE |
| 22 | Maria Caterina Manca | *Member* | SIGERIS |
| 23 | Andrea Vania | *Member* | SIMA |
| 24 | Vincenzo Provenzano | *Member* | SIMDO |
| 25 | Gerardo Medea | *Member* | SIMG |
| 26 | Primiano Iannone | *Member* | SIMI |
| 27 | Andrea Pession | *Member* | SIMMESN |
| 28 | Emanuele Cereda | *Member* | SINPE |
| 29 | Simona Bo | *Member* | SINU |
| 30 | Samir Sukkar | *Member* | SINUC |
| 31 | Luca Busetto | *Member* | SIO |
| 32 | Paolo Sbraccia | *Member* | SIO |
| 33 | Silvio Buscemi | *Member* | SIO |
| 34 | Cristina Segura Garcia | *Member* | SIPA |
| 35 | Vincenzo Pilone | *Member* | SIPAD |
| 36 | Patrizia Todisco | *Member* | SISDCA |
| 37 | Maurizio Santomauro | *Member* | SIT |
| 38 | Giovanni Docimo | *Member* | SIUEC |
| 39 | Matteo Monami | *Methodologist* | - |
| 40 | Federico Spandonaro | Economist | - |

ACOI - ASSOCIAZIONE CHIRURGHI OSPEDALIERI ITALIANI; ADI - ASSOCIAZIONE ITALIANA DI DIETETICA E NUTRIZIONE; AGIPPSA – ASSOCIAZIONE GRUPPI ITALIANI PSICOTERAPIA PSICOANALITICA DELL’ADOLESCENZA; - ASSOCIAZIONE ITALIANA DISTURBI DELL’ALIMENTAZIONE E DEL PESO; AIP - ASSOCIAZIONE ITALIANA DI PSICOLOGIA; AMD Associazione Medici diabetologi; AME - ASSOCIAZIONE MEDICI ENDOCRINOLOGI; ANIED - ASSOCIAZIONE NAZIONALE INFERMIERI IN ENDOCRINOLOGI; ANSISA - ASSOCIAZIONE NAZIONALE SPECIALISTI IN SCIENZA DELL'ALIMENTAZIONE; ASAND - ASSOCIAZIONE SCIENTIFICA ALIMENTAZIONE NUTRIZIONE E DIETETICA; CNAI - CONSOCIAZIONE NAZIONALE ASSOCIAZIONI INFERMIERE/I; GISEG - GRUPPO ITALIANO SALUTE E GENERE; SICE - SOCIETà ITALIANA CHIRURGIA ENDOSCOPICA.E NUOVE TEECNOLOGIE; SICOB - SOCIETA' ITALIANA DI CHIRURGIA DELL'OBESITA' E DELLE MALATTIE METABOLICHE; SICPRE - SOCIETA' ITALIANA DI CHIRURGIA PLASTICA RICOSTRUTTIVA ED ESTETICA; SID: SOCIETA' ITALIANA DI DIABETOLOGIA E DELLE MALATTIE DEL METABOLISMO; SIE - SOCIETA' ITALIANA ENDOCRINOLOGIA; SIED - SOCIETA' ITALIANA ENDOSCOPIA DIGESTIVA; SIGE - SOCIETA' ITALIANA DI GASTROENTEROLOGIA ED ENDOSCOPIA DIGESTIVA; SIGERIS - SOCIETA' ITALIANA GESTIONE RISCHIO IN SANITA'; SIMDO - SOCIETA' ITALIANA METABOLISMO DIABETE OBESITA'; SIMG - SOCIETA' ITALIANA DI MEDICINA GENERALE E DELLE CURE PRIMARIE; SIMI - SOCIETA' ITALIANA DI MEDICINA INTERNA; SIMMESN - SOCIETA' ITALIANA PER LO STUDIO DELLE MALATTIE METABOLICHE EREDITARIE.
